# CVB3-induced P62 cleavage promotes ferroptosis through the NRF2/GPX4 axis and facilitates viral replication

**DOI:** 10.3389/fmicb.2025.1675667

**Published:** 2025-11-21

**Authors:** Feng He, Zhuo Liu, Miao Feng, Dingding Cao, Sen Li, Xuelai Liu, Yiting Jiang, Xiaoyu Yi, Zhewei Liu, Hailan Yao

**Affiliations:** 1 Department of Biochemistry and Immunology, Capital Center for Children’s Health, Capital Medical University, Capital Institute of Pediatrics, Beijing, China; 2 Department of Surgery, Capital Center for Children’s Health, Capital Medical University, Capital Institute of Pediatrics, Beijing, China; 3 Department of Physilogy, Capital Center for Children’s Health, Capital Medical University, Capital Institute of Pediatrics, Beijing, China

**Keywords:** CVB3, ferroptosis, viral replication, P62, GPx4, selenium

## Abstract

**Introduction:**

Coxsackievirus B3 (CVB3) represents a major etiological agent of viral myocarditis, whose propagation within host organs results in substantial tissue injury. The molecular pathways through which CVB3 exerts its pathological effects, particularly its connection to ferroptosis-a regulated cell death modality characterized by iron-dependent lipid peroxidation-remain incompletely defined.

**Methods:**

We employed a combination of *in vitro* and *in vivo* models of CVB3 infection. Molecular techniques including immunoblotting, gene manipulation, and viral assays were used to investigate the proteolytic processing of the selective autophagy receptor P62 and its functional consequences. The role of the KEAP1/NRF2/GPX4 axis was examined using a non-cleavable P62 mutant. Furthermore, the therapeutic potential of a selenium-rich diet was evaluated in infected mice.

**Results:**

Our data showed that CVB3 replication induces the cleavage of P62 into distinct C- and N-terminal fragments. This event promotes the degradation of the transcription factor NRF2, leading to the downregulation of its target, GPX4, a key inhibitor of ferroptosis. Expression of a non-cleavable P62 mutant effectively stabilized the KEAP1/NRF2/GPX4 pathway and attenuated ferroptotic cell death in both cellular and mice models. Notably, GPX4 levels were not modulated by ubiquitination during infection. Supplementation with a selenium-rich diet, crucial for GPX4 synthesis, suppressed ferroptosis and improved survival rates in CVB3-infected mice.

**Discussion:**

This study identifies a novel mechanism whereby CVB3 exploits the cleavage of P62 to inactivate the KEAP1/NRF2/GPX4 axis, thereby driving ferroptosis and disease progression. These findings highlight the therapeutic potential of restoring P62 function and supplementing selenium to alleviate CVB3-induced pathogenesis.

## Highlights

- The CVB3 cleavage of the P62 protein participates in the KEAP1/NRF2/GPX4 axis and modulates CVB3-induced ferroptosis.
- Exogenous P62 protein supply partially rescues CVB3-induced ferroptosis.
- Selenium alleviates CVB3-induced myocarditis through the inhibition of ferroptosis.

## 1 Introduction

Coxsackie B group virus infections can cause a variety of diseases, from mild clinical symptoms such as flu-like illness and epidemic pleurodynia to severe conditions including acute and chronic myocarditis, congestive heart failure, and rhabdomyolysis, thereby posing a significant threat to human health (Xiao et al., 2022; Li et al., 2025; Bastea Li et al., 2024). One of the major members of the Coxsackie B group viruses is the Coxsackie B group type III (CVB3) virus (He et al., 2019; He et al., 2015; He et al., 2014), which primarily causes acute and chronic viral myocarditis and related diseases; however, there are still no specific preventive or therapeutic approaches in clinical practice (Yu et al., 2022). During the acute infection phase, CVB3 directly damages the structure and function of the myocardium (Liu et al., 2000; Belkaya et al., 2017), leading to myocardial damage by activating and maintaining the immune response (He et al., 2014). Therefore, inhibiting viral replication in the target organs during the early stages of infection is crucial for managing CVB3 infections (Xiao et al., 2022; Kelm et al., 2025). CVB3 infection also leads to pathological processes such as ferroptosis, necrosis, apoptosis, and autophagy in cells (He et al., 2019; Kang et al., 2025; Qiu et al., 2024; Yi et al., 2022), which contribute to its release and replication, further exacerbating myocardial damage.

Ferroptosis is a form of regulated cell death driven by iron ions and lipid peroxidation. It is characterized by oxidative damage to the phospholipids of long-chain polyunsaturated fatty acids found in cellular and organellar membranes (Fang et al., 2023; Mishima et al., 2025) This process is implicated in diverse physiological and pathological contexts, including carcinogenesis, inflammatory responses, neurodegenerative disorders, stroke, and cardiovascular diseases (Mishima et al., 2025; Sugimoto et al., 2025; Liu et al., 2025). Previous studies have shown that CVB3 infection could induce ferroptosis. Yi L. et al. further showed that CVB3 infection induces ferroptosis through the SP1/TFRC/GPX4 pathway and that the ferroptosis-associated gene transferrin receptor (TFRC) contributes to CVB3-induced ferroptosis (Yi et al., 2022; Yi et al., 2024). In their study, CVB3-induced ferroptosis was shown to promote viral replication. Inhibition of SP1 partially restored GPX4 expression and inhibited ferroptosis; however, viral replication caused by ferroptosis was not altered by SP1 depression. These findings suggest that additional pathways, beyond the established regulators, contribute to the dysregulation of GPX4 and the progression of ferroptosis during CVB3 infection. Previous studies have demonstrated that the NRF2/GPX4 axis plays a role in the ferroptosis process. In sepsis-associated acute kidney injury, suppression of NRF2 expression leads to a reduction in GPX4 levels, consequently increasing Fe^2+^ and reactive oxygen species (ROS) generation, which exacerbates ferroptosis and accelerates AKI progression (Zheng et al., 2024). Similarly, during bovine viral diarrhea virus (BVDV, family *Flaviviridae*) infection, the virus downregulates cytosolic and mitochondrial GPX4 via the Nrf2/GPX4 pathway, leading to lethal lipid peroxidation and promoting cellular ferroptosis (Li et al., 2024). These findings demonstrate that the NRF2/GPX4 axis plays an important regulatory role in ferroptosis. In our study, we showed that the KEAP1/NRF2/GPX4 axis is involved in the CVB3-induced ferroptosis process and that P62, which is located upstream and modulates expression of NRF2, could be cleaved by a CVB3 protease and contributes to the modulation of ferroptosis and viral replication through this axis. Exogenous supplementation of the mutant P62 protein can ultimately upregulate the expression of GPX4 by modulating NRF2 expression, thereby inhibiting CVB3-induced ferroptosis and suppressing viral replication. We further showed that the supplementation of selenium contributes to the attenuation of CVB3 by promoting the upregulation of GPX4 and inhibiting ferroptosis.

## 2 Materials and methods

### 2.1 Virus and animals

The CVB3m strain was a gift from Dr. Yang at the University of British Columbia, Canada, and was passaged in HeLa cells (Liu et al., 1999). The strain was preserved in our laboratory. The CVB3 sequence corresponds to accession number M33854.1 in the NCBI database. BALB/c male mice (6–8 weeks old), which are susceptible to CVB3 infection and commonly used to establish murine models of viral myocarditis, were purchased from the Institute of Laboratory Animal Sciences of China (Beijing, China). This animal study was approved by the Review Board of the Capital Institute of Pediatrics (Beijing, China) under the ethical code DWLL2024004.

### 2.2 Bioinformatics analysis

Global transcript profiling of hearts from the CVB3-infected mice was conducted using the Arraystar Mouse Microarray V3.0, as previously described (Zhang et al., 2013), with hearts from MOCK-infected mice serving as controls. Differently expressed genes in the infected group were identified for further analysis. Differently expressed genes with a fold change greater than 2 and an FDR < 0.01 were selected. KEGG pathway enrichment analysis was conducted using the clusterProfiler R package. Differently expressed genes were mapped to the KEGG database^1^ to identify significantly enriched pathways (Fisher’s exact test with Benjamini–Hochberg correction, *p* < 0.05). Visualization was performed using ggplot2^2^. Genes with direct or indirect interactions with GPX4 were filtered, and protein–protein interaction networks were generated using the STRING database^3^. The cutoff threshold (confidence score) was set at >0.4.

### 2.3 Mitochondrial morphology observation

The morphology of the mitochondria in myocardial tissue was observed using a JEM 1400 transmission electron microscope (Japan). The mice were anesthetized via intraperitoneal (i.p.) injection of ketamine (60 mg/kg, Shanghai Macklin Biochemical Corp, Shanghai, China) and xylazine (10 mg/kg, Shanghai Macklin Biochemical Corp, Shanghai, China), followed by euthanasia through cervical dislocation. The mice’s heart tissue was fixed in 2.5% glutaraldehyde for 12 h, washed, and post-fixed in 1% osmium tetroxide at room temperature for 2 h. The tissue was then dehydrated using a graded ethanol series and subsequently embedded in pure epoxy resin at 40 °C for 12 h. After embedding, thin sections of the tissue were cut and stained with uranyl acetate and lead citrate, and they were observed under an electron microscope.

### 2.4 Viral plaque assay

Viral titers were determined by plaque assay as described previously (Xiao et al., 2022). Briefly, HeLa cells (8 * 10^5^ cells/well) were seeded into six-well plates and incubated at 37 °C for 24 h. The cells were then washed twice with PBS, overlaid with 200 μL of a 1:10 diluted virus-containing supernatant, and subsequently overlaid with 2 mL of sterilized soft Bacto agar-MEM. Thereafter, the cells were incubated at 37 °C for 72 h and fixed with Carnoy’s fixative for 30 min, followed by staining with 1% crystal violet. Consequently, plaques were counted, and viral titers (PFU/ml) were calculated as described (Xiao et al., 2022).

### 2.5 Protein detection

HeLa cells and human cardiac myocytes (AC16) were used in this study. The cells were infected with CVB3 at a multiplicity of infection (MOI) of 10, while the mice were infected with 1.0*10^5^ plaque-forming units of CVB3, which corresponds to LD50 for mice. Cells or homogenized mouse heart tissues were collected at the indicated time point and lysed in RIPA buffer (Kangwei, Beijing, China). Antibodies for detecting P62, P62-c terminal, NRF2, GPX4, HO-1, Keap1, and *β*-actin were purchased from Cell Signaling Technology (USA). Western blotting was conducted as described previously (Xiao et al., 2022).

### 2.6 Measurement of serum cytokines

Serum samples from the CVB3-infected mice were separated by centrifugation and stored at −80 °C until analysis. The samples were diluted appropriately with the provided assay diluent. Cytokine levels in the serum were measured using the Cytometric Bead Array kit (CBA, BioLegend, USA) according to the manufacturer’s instructions.

### 2.7 Co-IP

Tissues or cells were lysed using lysis buffer and incubated with antibodies overnight at 4 °C, then they were mixed with 30 μL of protein A/G magnetic beads, which bind to antibodies to form bead–protein–antibody complexes, followed by a 2 h incubation at 4 °C. A magnetic rack was used to capture the beads, while unbound proteins were discarded. Subsequently, the beads were washed six times with wash buffer at 4 °C and boiled, followed by immunoblotting analysis.

### 2.8 Fe^2+^ detection

The content of iron (Fe^2+^) was measured according to the manufacturer’s instructions (DOJINDO, Japan). HeLa cells or myocardial tissues were homogenized in a buffer containing four volumes of the iron content determination solution. Insoluble materials were removed by centrifugation at 16,000 g at 4 °C, and the supernatant was then mixed with an equal volume of water, an equal volume of Solution B, and 20 times the volume of Solution A. The mixture was incubated in the dark at room temperature for 40 min with 200 μL/well of iron probe solution. The OD value was measured at a wavelength of 590 nm.

### 2.9 Reactive oxygen species (ROS), malondialdehyde (MDA), glutathione (GSH), and heme oxygenase 1 (HO-1) detection

The content of ROS was measured using the Reactive Oxygen Species Assay Kit according to the manufacturer’s instructions (Beyotime, China). Briefly, 1 × 10^4^ freshly isolated heart cells or HeLa cells were resuspended in 1 mL PBS and stained with 100 μL of 10 μM DCFH-DA dye in the dark, followed by gentle inverting and mixing every 5 min. After 25 min, the cells were centrifuged, and the supernatant was discarded. The plates were then washed twice with DMEM and resuspended in 0.1 mL PBS, followed by measurement of the OD value at 470 nm. MDA was measured using the Lipid Peroxidation MDA Assay Kit according to the manufacturer’s instructions (Beyotime, China). HeLa or mouse heart cells were homogenized and centrifuged at 12,000 × *g* for 10 min at 4 °C. The supernatant was collected to determine the protein concentration using the BCA method. The supernatant was also mixed with thiobarbituric acid detection solution and used to measure the OD value at 532 nm. GSH in cell lysates or tissue lysates was measured using a GSH Assay Kit (Beyotime, China) following the manufacturer’s protocol. HO-1 in cell lysates was measured using a HO-1 Assay Kit (Bio-Lab Technology, China) following the manufacturer’s protocol.

### 2.10 P62 adeno-associated virus

Adeno-associated viruses were constructed to contain the P62 mutant expression plasmid. A viral suspension of 2.47 × 10^13^ particles/mL was set as the initial concentration. Exactly 20 μL of the initial virus was injected intravenously via the tail vein, with each mouse receiving a total viral load of 5 × 10^11^ particles. At 21 days post-injection, the CVB3 challenge was performed.

### 2.11 Selenium administration

The treated HeLa cells were cultured in MEM containing 50 nM sodium selenite, while the MOCK group was cultured in medium with a normal selenium level. Furthermore, 4-week-old BALB/c mice were fed a normal or selenium-enriched diet at 0.02 mg/mouse. Sodium selenite was mixed with drinking water at a concentration of 1 mg/250 mL based on a daily water intake of 5 mL per mouse for 2 weeks and administered via intraperitoneal injection alongside the CVB3 strain at an LD50 dose. During the infection period, sodium selenite was administered via intragastric gavage at a dose of 0.02 mg/mouse per day. The protocols for *in vitro* selenium-enriched culture and selenium-supplemented diets were adapted from Yao et al. (2021) and Jun et al. (2011). The mice were then euthanized on days 3, 5, and 7 post-infection under anesthesia, and their cardiac tissues were collected to measure viral titers and assess myocardial pathological damage.

### 2.12 Data analysis

All statistical analyses were performed using the SPSS 26.0 computer software program (SPSS, Inc., Chicago, IL). Survival was analyzed using the log-rank (Mantel-Cox) method. The significance of variability among the experimental groups was determined using the Mann–Whitney U test. All differences were considered statistically significant at a *p*-value of < 0.05. Figures were drawn using GraphPad Prism (version 9.5.1).

## 3 Results

### 3.1 CVB3 infection-induced ferroptosis in the mouse model

**Figure 1:**
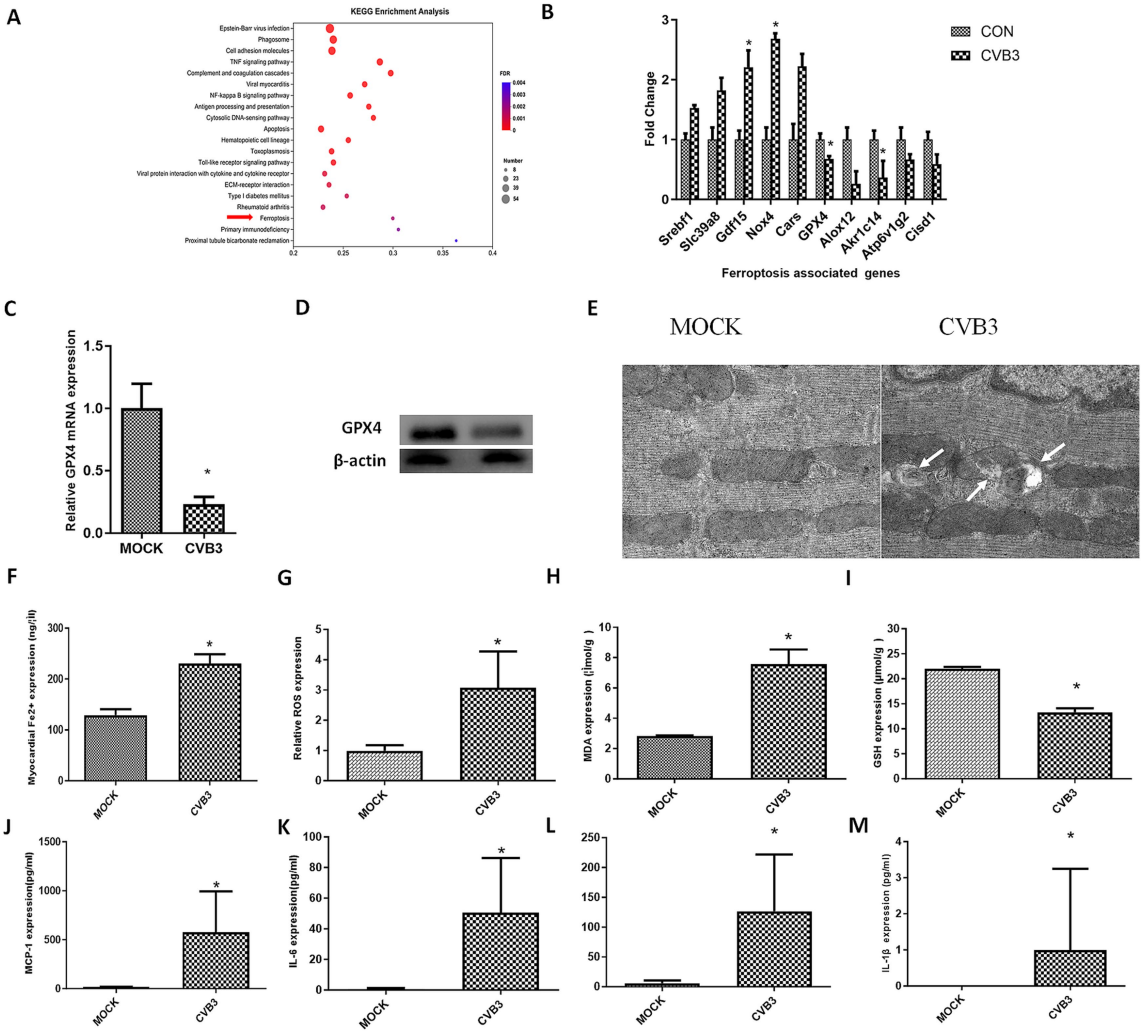
CVB3 infection induces ferroptosis in a virus-induced myocarditis mouse model. BALB/c mice were infected intraperitoneally with 1.0*10^5^ plaque-forming units of CVB3, corresponding to the LD50 for mice. MOCK-infected mice received intraperitoneal injections of DMEM and were included as the MOCK group. **(A)** The top 20 KEGG-enriched pathways, including the ferroptosis signaling pathway. **(B)** Differently expressed genes associated with ferroptosis are shown. The data used for **(A,B)** were obtained from our previous study (Zhang et al., 2013). **(C)** The mice were anesthetized and sacrificed for heart collection. The tissues were then homogenized for RNA extraction, and real-time quantitative PCR detection of GPX4 was conducted. **(D)** GPX4 expression was detected in the hearts of the CVB3-infected mice. **(E)** The mice were anesthetized and sacrificed for heart collection. Morphology of mitochondria in myocardial tissue was observed. Representative electron microscopy images indicating mitochondrial membrane ruptures (white arrows), reduced volume, and decreased cristae. **(F)** Contents of ferrous ions in myocardial homogenate tissue. Reactive oxygen species (ROS) **(G)**, malondialdehyde (MDA) **(H)**, and glutathione (GSH) **(I)** in the myocardial homogenate tissue are shown. Inflammatory cytokines in the serum of the infected mice were also detected: MCP-1 **(J)**, IL-6 **(K)**, IFN-*β* **(L)**, and IL-1β **(M)**. * indicates *p* < 0.05. A total of six mice were used in the experiment.

In this study, we performed transcriptional analysis of the heart in a mouse model on day 5 post-CVB3 infection at a dose of 1x10E5 PFU (LD50), corresponding to the acute infection stage. Significantly different genes and enriched pathways with an FDR of < 0.01 were selected following KEGG analysis. Among these, the top 20 pathways with the highest rich factor values, representing the number of differentially expressed genes (DEGs) in each pathway, were plotted. The ferroptosis signaling pathway was also enriched (Figure 1A). After CVB3 infection, ferroptosis-associated genes were altered (Figure 1B). Genes that promote ferroptosis, such as Gdf15, Nox4, and Srebf1, were upregulated, while genes that inhibit ferroptosis, such as GPX4, Akr1c14, and Cisd1, were downregulated, indicating that CVB3 caused ferroptosis. (Figure 1B). GPX4, which is critically involved in the occurrence of ferroptosis, was validated using real-time quantitative PCR and western blotting, confirming the downregulation of the gene in the CVB3-infected group (Figures 1C,D).

Ferroptosis is characterized by mitochondrial condensation, which is manifested by morphological alterations, including reduced volume, increased membrane density, and diminished cristae in cellular mitochondria. Mitochondrial morphology in the myocardial tissue of the CVB3-infected mice was observed using electron microscopy, showing typical ferroptosis in the mitochondria of cardiomyocytes (Figure 1E) and increased cellular Fe^2+^ levels (Figure 1F). ROS (Figure 1G) and MDA (Figure 1H), which are terminal products of lipid peroxidation and established biomarkers of oxidative damage, were also enhanced in the CVB3-infected mice, while GSH as an important antioxidant was decreased after CVB3 infection (Figure 1I). We also detected inflammatory cytokines such as MCP-1 (Figure 1J), IL-6 (Figure 1K), IFN-*β* (Figure 1L), and IL-1β (Figure 1M) in the serum of the CVB3-infected mice, with the results showing that these cytokines were highly expressed post-infection.

### 3.2 Coxsackie B group type III virus infection induces ferroptosis through the NRF2/GPX4 axis

**Figure 2:**
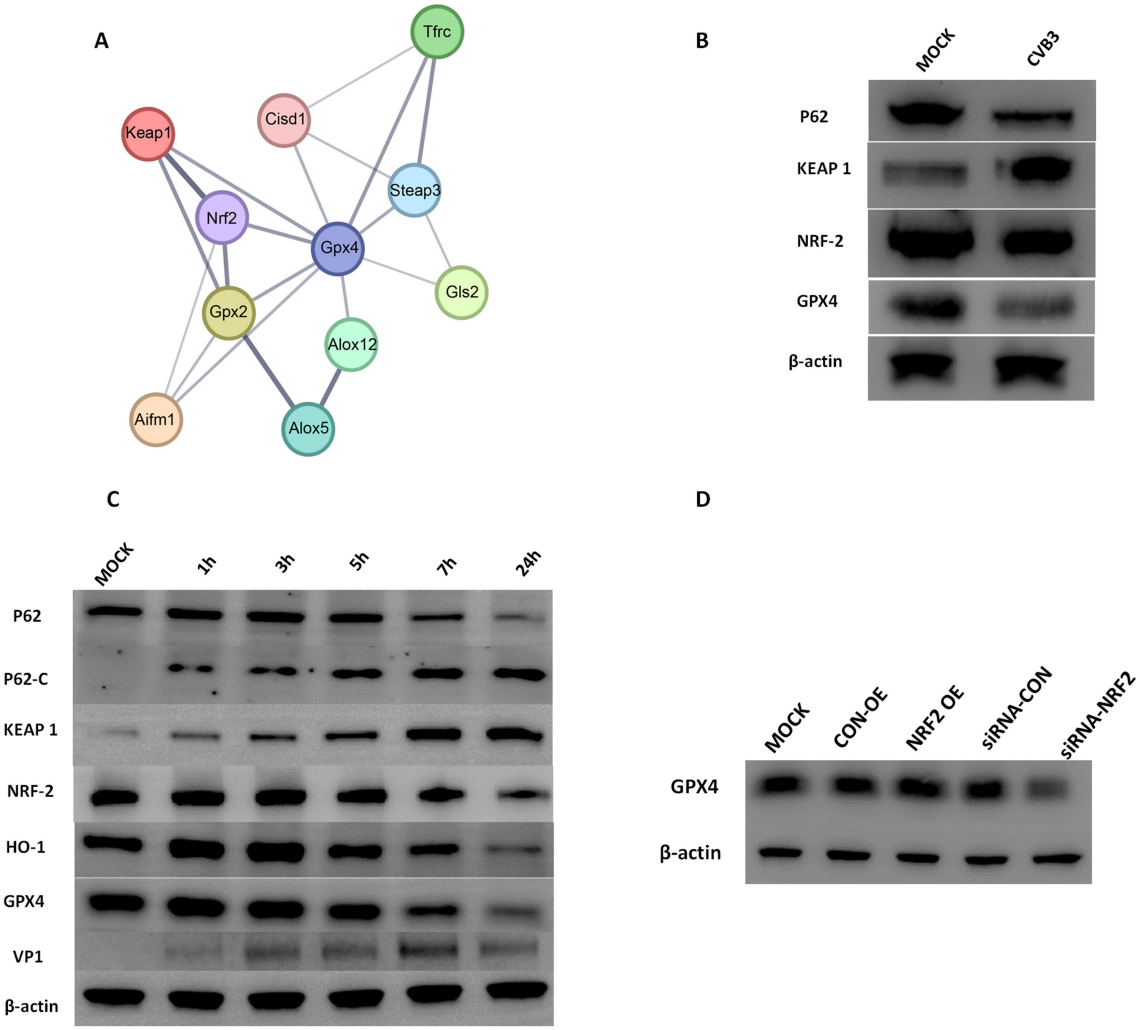
**(A)** Protein–protein interaction networks of downregulated and differentially expressed genes directly or indirectly related to GPX4. The thickness of the lines represents the credibility of the interaction between proteins (comprehensive score value). **(B)** CVB3 infection altered the expression of KEAP1, NRF2, and GPX4. AC-16 cells were infected with CVB3 at an MOI of 5, and profiling of total P62, KEAP1, NRF2, and GPX4 proteins was detected. β-actin is shown as a loading control. The results represent three independent tests. **(C)** CVB3 infection cleaved the P62 protein and altered the expression of KEAP1, NRF2, and GPX4. HeLa cells were infected with CVB3 at an MOI of 10, and the time course of profiling of total P62, cleaved P62, and the expression of KEAP1, NRF2, and GPX4 proteins was detected. β-actin is shown as a loading control. The results represent three independent tests. **(D)** Detection of GPX4 expression following NRF2 alteration during the CVB3 infection process. NRF2 was exogenously expressed or inhibited, followed by CVB3 infection at an MOI of 10. The expression of GPX4 was detected using western blotting 24 h post-CVB3 infection. OE indicating overexpression, * indicates *p* < 0.05. A representative result from three independent experiments is shown.

Bioinformatics analysis was conducted to identify potential signaling pathways associated with the downregulation of GPX4 during CVB3 infection. By analyzing all downregulated genes during CVB3 infection, a protein–protein interaction network was constructed to examine all genes that may have direct or indirect interactions with GPX4. The analysis revealed a strong interaction between the KEAP1/NRF2 pathway and GPX4 (Figure 2A).

To examine whether the NRF2/GPX4 axis is involved in the CVB3 infection process, we infected AC16 cells with CVB3 at an MOI of 5 for 12 h, and the results showed that KEAP1 was enhanced compared to the CON group, while NRF2 and GPX4 were decreased (Figure 2B). To further observe the temporal changes in NRF2/GPX4 signaling pathways caused by CVB3 infection, HeLa cells, which are wildly used to study the mechanisms of CVB3 infection, were infected with CVB3 at an MOI of 10, as described in a previous study (Yi et al., 2022). Continuous expression of NRF2 and GPX4 proteins was detected, with their expression gradually decreasing as the time of infection increased (Figure 2C). KEAP1, which is located upstream and negatively modulates the expression of NRF2, was increased (Figure 2C). While HO-1, which is located downstream of NRF2 and modulates the expression of GPX4, was significantly decreased in CVB3-infected HeLa cells. To further validate the regulatory function of NRF2 on GPX4, we constructed and transfected an NRF2 expression plasmid into HeLa cells before CVB3 infection. GPX4 showed higher expression in CVB3-infected HeLa cells than in the MOCK group. Inhibition of NRF2 was also performed using specific siRNA, which resulted in decreased GPX4 levels, as expected, confirming that the NRF2/GPX4 axis is involved in the process of CVB3 infection and that NRF2 could modulate GPX4 (Figure 2D).

### 3.3 CVB3 modulates ferroptosis in a P62-dependent manner

**Figure 3:**
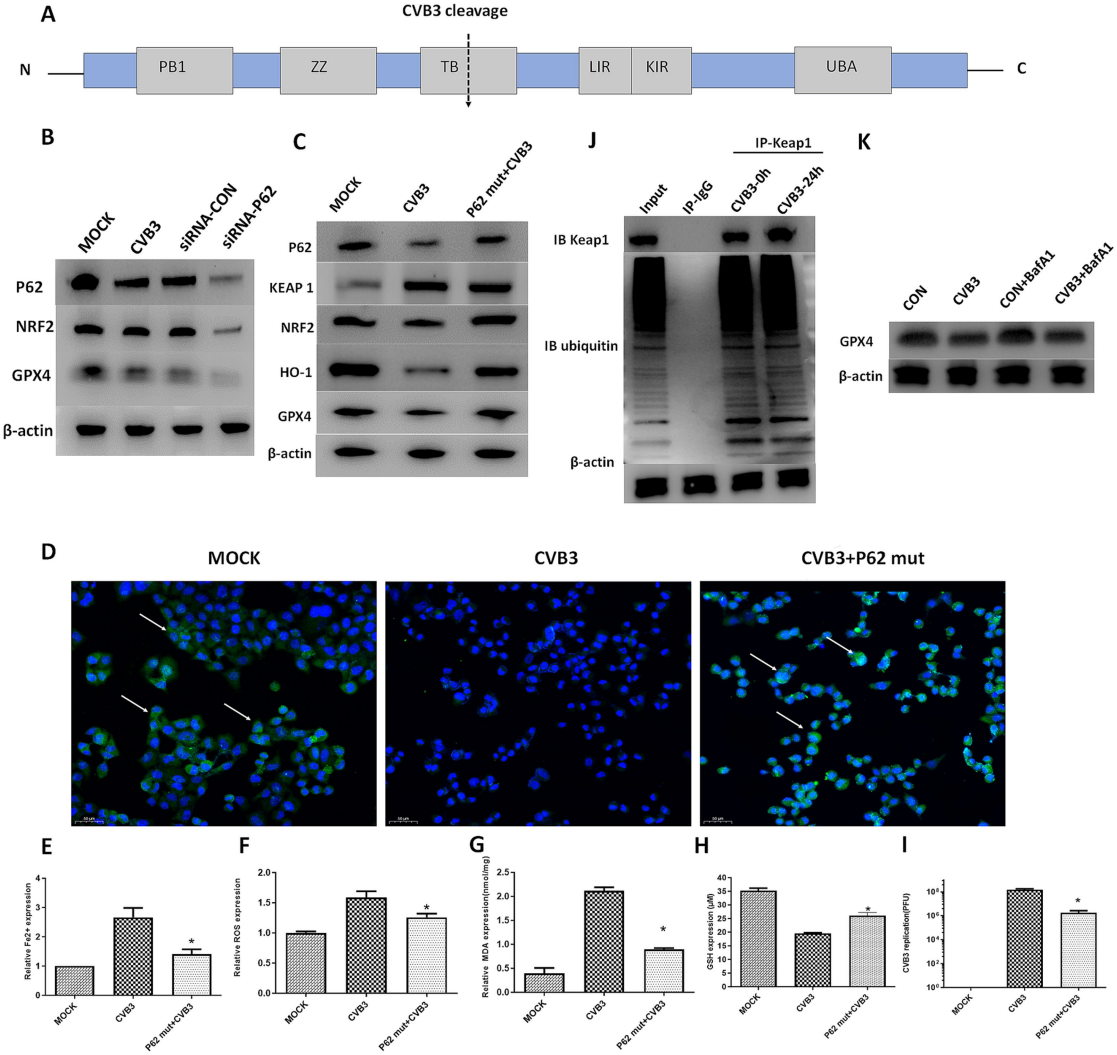
P62 participates in the KEAP1/NRF2/GPX4 axis and modulates ferroptosis in the CVB3 infection process. The P62 mutant was exogenously expressed, followed by CVB3 infection at an MOI of 10. **(A)** Schematic illustration of the P62 polyprotein. The arrow depicts the cleavage site of virus-encoded proteinases. **(B)** Detection of NRF2 and GPX4 in the CVB3-infected mice with silenced P62 at an MOI of 10 using western blotting 24 h after viral infection. **(C)** Detection of the KEAP-1/NRF2/GPX4 axis after P62 mutant overexpression in the CVB3 infection process using western blotting 24 h after infection. **(D)** Detection of NRF2 expression in the cell nucleus after P62 inhibition during the CVB3 infection process using immunofluorescence. Detection of ferroptosis-associated makers, including Fe^2+^ **(E)**, ROS **(F)**, MDA **(G)**, and GSH **(H)**, in HeLa cells after exogenous P62 expression during the CVB3 infection process. **(I)** Detection of CVB3 replication post-P62 overexpression 24 h after viral infection. **(J)** Cells were lysed 24 h post-CVB3 infection. Total KEAP1 protein and its interacting proteins were pulled down using an antibody against KEAP1, followed by immunoblotting (IB) with antibodies against KEAP1 and ubiquitin. HeLa cells without infection were used as the MOCK control. **(K)** HeLa cells were treated with 20 nM of BafA1 before CVB3 infection, and GPX4 expression was then detected. * Indicates *p* < 0.05, and a representative result from three independent experiments is shown.

Since the selective autophagy receptor and ubiquitin sensor protein, P62, plays an important role in the process of ferroptosis in various diseases, such as tumors and inflammatory diseases, we determined the expression of P62 in CVB3-infected HeLa cells at different time points. The expression of the P62 protein gradually and significantly decreased 24 h after CVB3 infection (Figures 2B,C). In addition, P62 can be cleaved into two parts by the 2A protease of CVB3 at glycine 241 (G241) to create N- and C-terminal ends (Figure 3A). In this study, P62 was cleaved, and its C-terminal fragment gradually increased due to the decreasing process of P62 (Figure 2C). In contrast, no change in the N-terminal fragment was detected, since this end is easily exposed, making its expression undetected. To validate the function of P62 in modulating the expression of the KEAP-1/NRF2/GPX4 axis, P62 in HeLa cells was silenced using specific siRNA and infected with CVB3. NRF2 and GPX4 decreased in the P62-silenced group compared to the MOCK group, indicating that P62 modulates GPX4 through KEAP1 and NRF2 (Figure 3B).

Since P62 confers protection against ferroptosis in the cell, we determined its protective function on the NRF2/GPX4 axis in HeLa cells by constructing a P62-mut plasmid expressing a mutant protein of P62 at position 241 (P62-G241E) that prevents cleavage by the viral 2A protein but retains the full functionality of intact P62. The plasmid was used to transfect the HeLa cells 48 h before CVB3 infection. The results showed the upregulation of NRF2, GPX4, and HO-1 and the downregulation of KEAP1 after P62 transfection, as expected (Figure 3C). In normal physiological conditions, NRF2 and KEAP1 bind together, where KEAP1 inhibits the activity of NRF2, while under oxidative stress, the KEAP1–NRF2 complex dissociates, leading to the translocation of NRF2 into the nucleus. The immunofluorescence results showed that NRF2 expression in the nucleus was lower in CVB3-infected cells compared to the MOCK-infected group (Figure 3D). Ferroptosis-associated Fe2 + (Figure 3E), ROS (Figure 3F), and MDA (Figure 3G) levels were inhibited, GSH was upregulated (Figure 3H), and CVB3 replication was reduced (Figure 3I), indicating that the upregulation of P62 could inhibit virus-induced ferroptosis and viral replication.

Considering that P62 is mostly involved in the autophagy process of target proteins by interacting with ubiquitinated target proteins, we aimed to investigate whether P62 regulates GPX4 through the autophagy-dependent modulation of KEAP1, a direct interacting protein of P62, during CVB3 infection. The results showed that KEAP1 was not ubiquitinated during CVB3 infection, indicating that its expression could not be regulated by P62 via autophagy (Figure 3J). We further exogenously added the late-stage autophagy inhibitor Bafilomycin A1 (BafA1) to inhibit cellular autophagy function, and the results showed that GPX4 expression was not affected (Figure 3K).

### 3.4 P62 mutant overexpression attenuates CVB3-induced ferroptosis *in vivo*

**Figure 4:**
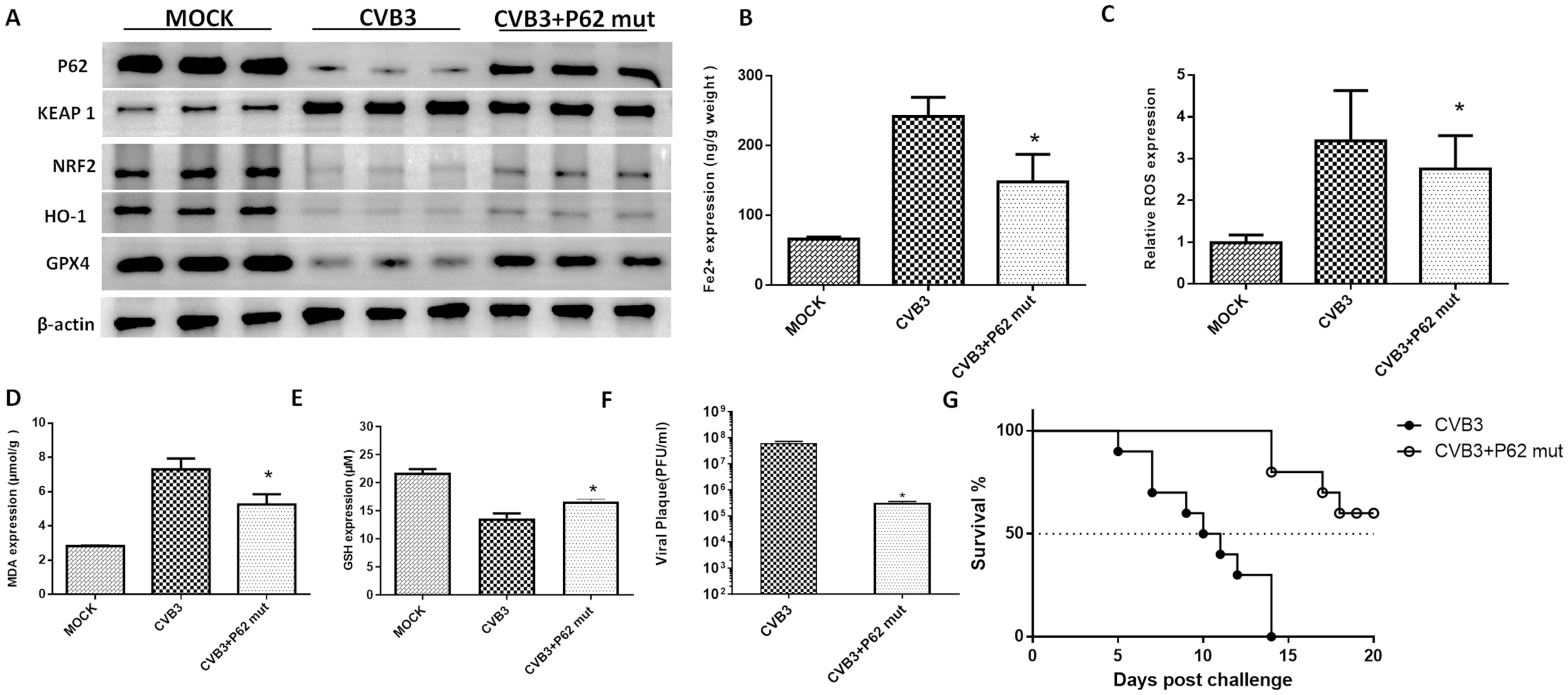
P62 protein supplementation inhibits ferroptosis and viral replication *in vivo*. **(A)** Detection of proteins in the KEAP-1/NRF2/GPX4 axis after the overexpression of P62 in the CVB3-infected mice using western blotting 5 days post-infection. P62 was overexpressed in the mice using an AAV containing the P62 gene, followed by CVB3 infection at a PFU of 10^5^, which corresponds to the LD50 for mice. Detection of ferroptosis-associated makers after P62 overexpression and supplementation, including the contents of Fe^2+^ **(B)**, ROS **(C)**, MDA **(D)**, and GSH **(E)** in the heart tissue 5 days post-CVB3 infection. **(F)** Titers of CVB3 in the heart tissue 5 days post-viral infection in the mice with overexpressed P62. **(G)** Survival of BALB/c mice post-CVB3 infection in the P62 overexpression group. * Indicates *p* < 0.05. A total of 10 mice were used in this experiment, and representative results of protein expression from three independent experiments are shown in **(A)**.

To validate the function of the P62-mut protein *in vivo*, the P62-mut plasmid was transduced into an adeno-associated virus and injected into the mice via the tail vein, followed by intraperitoneal injection of an LD50 dose of CVB3 21 days later. Subsequently, mouse hearts were collected at day 5 post-CVB3 infection. P62 was overexpressed in the heart of the mice, while GPX4 was upregulated. The upregulation of NRF2, GPX4, and HO-1 and the repression of KEAP1 were also observed (Figure 4A), consistent with our *in vitro* results. In addition, CVB3-induced ferroptosis reduced Fe^2+^ (Figure 4B), ROS (Figure 4C), MDA (Figure 4D), and CVB3 replication (Figure 4F) in the hearts of the mice, while GSH was highly expressed (Figure 4E), indicating the inhibition of ferroptosis by the overexpression of P62. Furthermore, P62-mut overexpression improved the survival rate and lifespan of the mice following LD50 CVB3 infection compared to the MOCK group (Figure 4G).

### 3.5 Selenium supplement upregulates GPX4 to inhibit CVB3-induced ferroptosis *in vitro* and alleviate the mice’s survival rate

**Figure 5:**
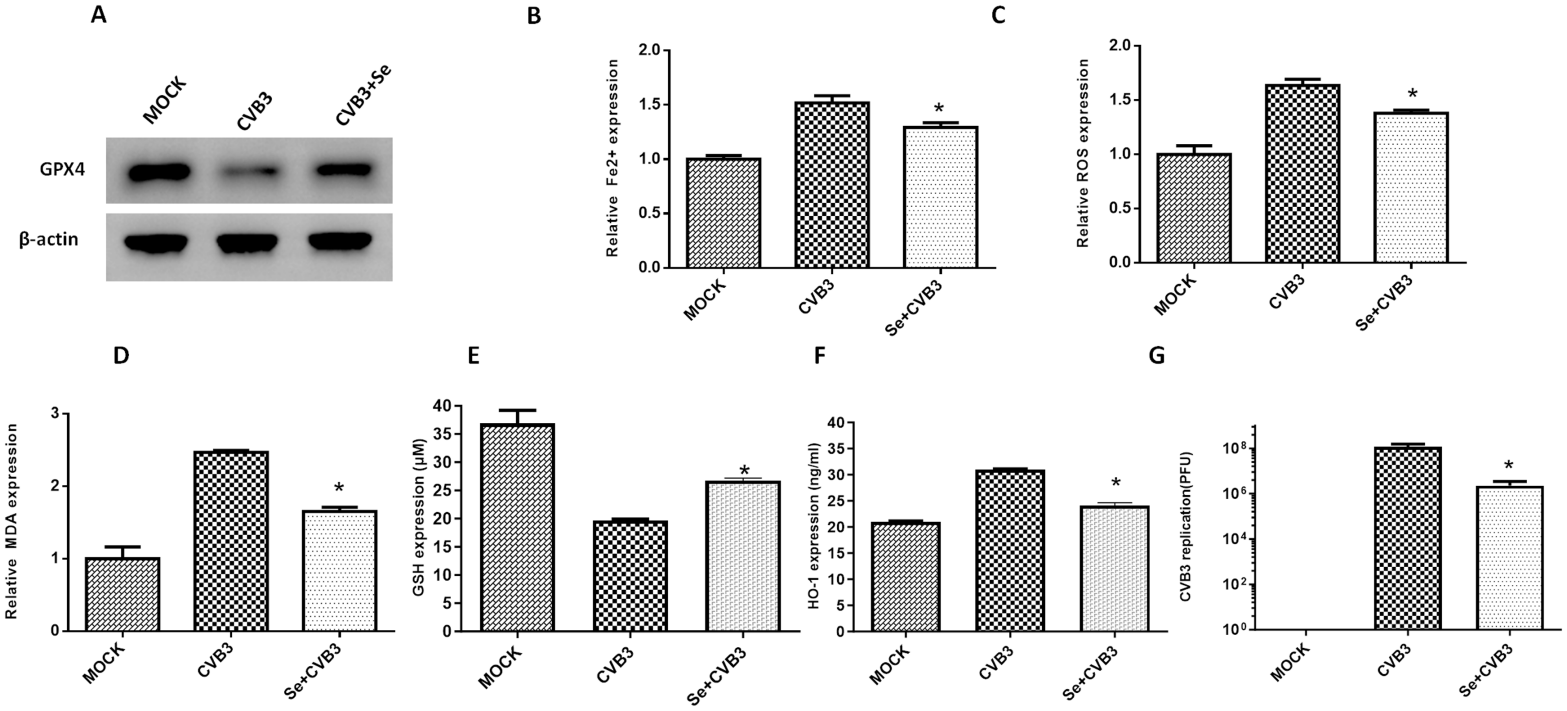
Selenium (Se) supplementation could alleviate ferroptosis *in vitro*. **(A)** Detection of GPX4 expression in HeLa cells supplemented with Se, followed by CVB3 infection, 24 h post-infection. The contents of ferroptosis-associated makers in HeLa cells, including Fe^2+^ **(B)**, ROS **(C)**, MDA **(D)**, GSH **(E)**, and HO-1 **(F)**, as well as CVB3 replication, were also detected post-Se supplementation 24 h after viral infection **(G)**. * Indicates *p* < 0.05. A total of three independent experiments were conducted, and a representative result of protein expression is shown in **(A)**.

CVB3 infection leads to ferroptosis in cells through the modulation of GPX4 proteins, and ferroptosis could promote viral replication. Studies also suggest that selenium has a certain inhibitory effect on viral replication, although the mechanism is not clear. Selenium is also an essential element in the human antioxidant system and is necessary for the synthesis of GPX4. In this study, we supplied HeLa cells with selenium before CVB3 infection and found higher expression of GPX4, attenuation of Fe^2+^ (Figure 5B), ROS (Figure 5C), and MDA (Figure 5D), and upregulation of GSH (Figure 5E) and HO-1 (Figure 5F). CVB3 replication inhibition was also observed in the selenium-treated group compared to the MOCK group (Figure 5G).

**Figure 6:**
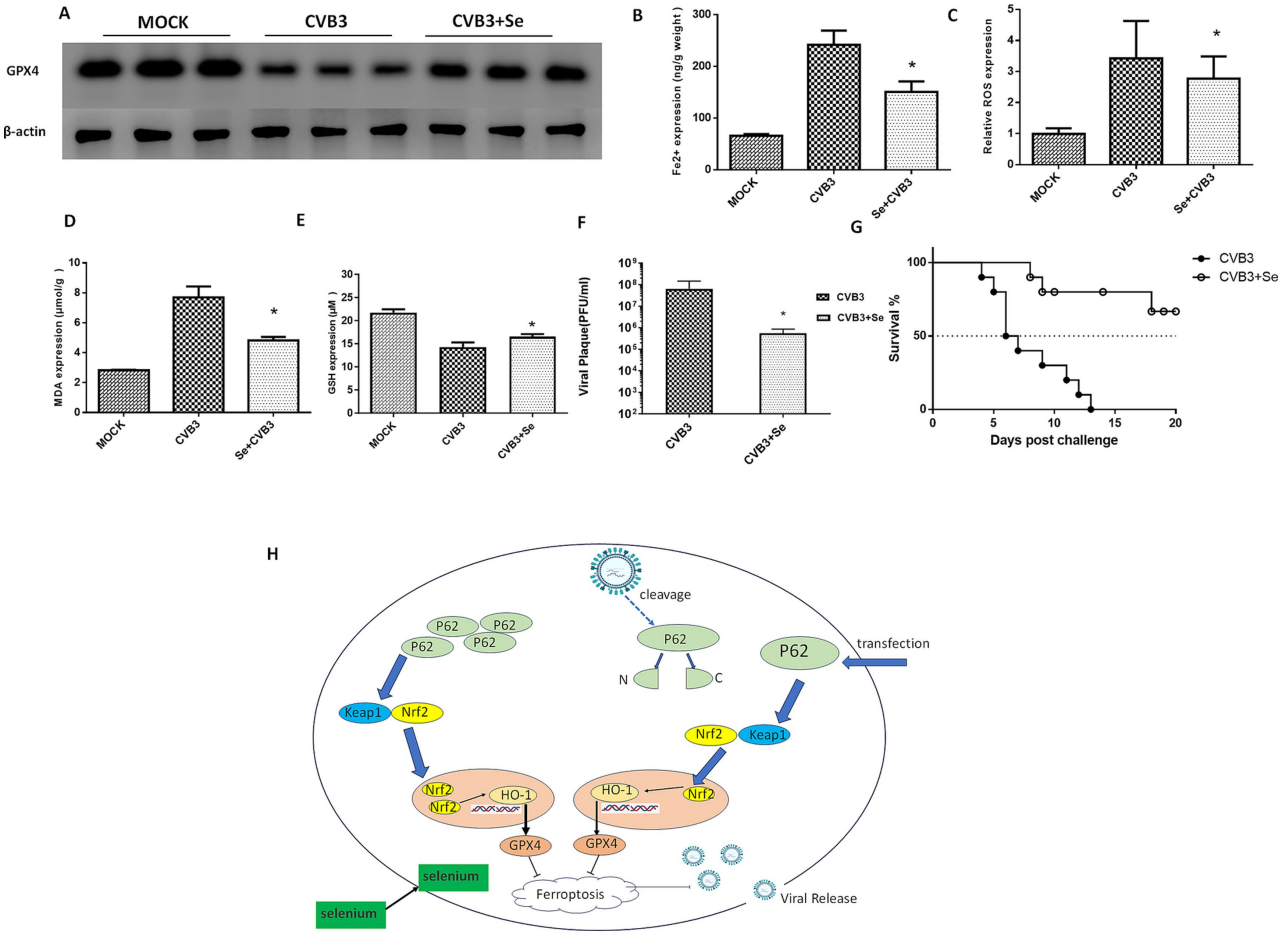
Selenium (Se) supplementation could inhibit ferroptosis and viral replication *in vivo*. **(A)** Detection of the GPX4 protein in the Se-supplemented and CVB3-infected mice 5 days post-infection. A selenium-enriched diet was provided to the mice for 7 days, after which they were infected with 10^5^ PFU of CVB3 at LD50. Contents of ferroptosis-associated makers in the heart tissues, including Fe^2+^ **(B)**, ROS **(C)**, MDA **(D)**, and GSH **(E)**, were detected post-Se and P62 supplementation 5 days after CVB3 infection. **(F)** CVB3 titers in the heart tissue 5 days post-viral infection in the mice that were fed a Se-supplemented diet. Virus titers (PFU/g) were normalized to the tissue mass. The virus titers represent the means ± SD of three independent experiments (*n* = 5 mice/group). **(G)** Survival of the BALB/c mice after CVB3 infection in the Se-supplemented diet. **(H)** Schematic illustration of this study. * indicates *p* < 0.05. A total of 10 mice were used in this experiment. Representative results of protein expression from three experiments are shown in **(A)**.

We subsequently examined the role of selenium in the ferroptosis process *in vivo* by feeding the mice a selenium-enriched diet for 4 weeks, followed by CVB3 infection. We found that the GPX4 protein in the mice heart was upregulated (Figure 6A). The expression of Fe^2+^ (Figure 6B), ROS (Figure 6C), and MDA (Figure 6D) was suppressed, while the expression of GSH (Figure 6E) was elevated in the selenium-treated group. Viral titers were also measured and found to be nearly two orders of magnitude lower than in the MOCK group (Figure 6F). Importantly, the mice exhibited a significantly increased survival rate and prolonged survival time in the selenium-treated group compared to the MOCK group (Figure 6G).

Finally, we concluded that CVB3 could induce ferroptosis by cleaving P62, thereby targeting the NRF2/GPX4 axis, and that supplementation with P62 or selenium could inhibit CVB3 replication by suppressing ferroptosis (Figure 6H).

## 4 Discussion

CVB3 is one of the most common and significant causative agents of myocarditis in humans, especially in infants and teenagers (Li et al., 2025), and there are still no specific preventive or therapeutic methods available in clinical practice. Virus infection induces ferroptosis, necrosis, pyroptosis, and autophagy in cells (He et al., 2019; Yi et al., 2022; Xin et al., 2014), which facilitate its release and replication and may cause severe inflammatory actions and further exacerbate myocardial damage. Among these pathological processes, ferroptosis results from an imbalance in cellular metabolism and redox equilibrium (Wang Y. et al., 2025; Dixon et al., 2012), while its occurrence requires iron overload, a large number of phospholipids with polyunsaturated fatty acid side chains in the cell, and impaired antioxidant function within the cell. On the other hand, the body has three proteins that inhibit ferroptosis, including cytoplasmic and mitochondrial glutathione peroxidase (GPX4), which reduces glutathione (GSH) to eliminate lipid peroxides such as ROS and suppresses ferroptosis. The other proteins are ferroptosis suppressor protein 1, located on the cell membrane where it reduces Coenzyme Q10 to Coenzyme Q10 H2, which scavenges lipid peroxyl radicals, and dihydroorotate dehydrogenase, located on the outer surface of the mitochondrial inner membrane, which decomposes and synthesizes dihydroorotic acid and orotic acid, both of which have inhibitory effects on ferroptosis (Nakamura et al., 2023; Mao et al., 2021; Baruah et al., 2023). Among these proteins, GPX4 is the central inhibitor of ferroptosis (Lancaster and Murphy, 2025), which is induced by CVB3. Yi L. et al. (1) showed that CVB3 infection induces ferroptosis in HeLa cells through the SP1/TFRC pathway, while the inhibition of SP1 can partially restore GPX4 expression and partially reverse ferroptosis. However, the viral titer caused by ferroptosis is not altered by SP1 depression. Therefore, we speculate that, in the process of CVB3-induced ferroptosis, there may be other pathways regulating GPX4 expression and participating in ferroptosis (He et al., 2015).

In this study, a CVB3-induced mouse model of myocarditis was constructed. Transcriptome analysis and bioinformatics analysis showed the enrichment of the ferroptosis pathway and the significant downregulation of its core inhibitor gene, GPX4. The interaction network for ferroptosis, constructed using altered CVB3-associated genes, indicated that the KEAP1/NRF2 signal pathway is associated with GPX4. NRF2 is the major regulator of the antioxidant defense system, which enhances cellular resistance to oxidative stress, thereby inhibiting ferroptosis (Yamane et al., 2022) and also plays a protective role in drug-induced cardiotoxicity and cardiac dysfunction (Yi et al., 2022). The KEAP1/NRF2 axis also participates in the ferroptosis process in ischemic flap survival (Sun et al., 2016), renal cell carcinoma (Chang et al., 2023), and pancreatic cancer (Feng et al., 2024). In this study, we showed that the KEAP1/NRF2 axis is also involved in the CVB3 infection process and that NRF2 is a transcription factor that upregulates the expression of the HO-1 protein, which, in turn, promotes the upregulation of the GPX4 protein. When the expression of NRF2 was inhibited, GPX4 was suppressed and ferroptosis was triggered.

The regulation of the KEAP1/NRF2 axis during CVB3 infection requires further study. The P62 protein, which can be directly cleaved by CVB3, caught our attention. P62, also known as sequestosome 1 (SQSTM1), is a selective autophagy receptor and ubiquitin (Ub) sensor protein located in the cytoplasm, where it binds and inhibits the function of KEAP1 (Martin et al., 2024). How P62 modulates the KEAP1/NRF2 axis has been elucidated in Alzheimer’s disease (Zhang et al., 2021), hypoxic–ischemic brain damage (Wang X. X. et al., 2025), and virus-induced ferroptosis in EBV-induced tumorigenesis (Yuan et al., 2022). However, how P62 is regulated during viral infection has not been elucidated. In this study, we showed that CVB3 cleaved P62, while the KEAP1/NRF2 axis was affected: KEAP1, which negatively regulates NRF2, was released from cleaved P62, and downregulated NRF2 attenuated GPX4 and further induced ferroptosis. Exogenous expression of the P62 mutant, which could not be cleaved by CVB3, partially reverses CVB3-induced ferroptosis through enhanced expression of NRF2 in the nucleus, which also elevates the expression of GPX4. This process could reverse ferroptosis and attenuate viral replication.

Since P62 is an autophagy receptor protein and a key molecule in selective autophagy, it primarily facilitates the autophagic degradation of target proteins by interacting with their ubiquitinated forms (Vargas et al., 2023). This led us to investigate whether the regulated expression of the KEAP1 protein during CVB3 infection is influenced by ubiquitination. We measured the ubiquitination level of KEAP1 following CVB3 infection and found that it remained unchanged, indicating that the role of P62 in regulating autophagy does not affect the KEAP1 protein. Furthermore, treatment with the late-stage autophagy inhibitor Bafilomycin A1 did not affect GPX4 expression, indicating that the ferroptosis process, triggered by reduced GPX4 expression after CVB3 infection, is not influenced by autophagy.

Studies have shown that selenium can inhibit viral replication. Acute hepatitis B patients were found to have significantly lower selenium levels compared to healthy individuals. *In vitro* studies have further demonstrated that supplementing selenium in cell culture media effectively suppresses hepatitis B virus replication (Cheng et al., 2016). A German study reported that survivors of SARS-CoV-2 infection had markedly higher blood selenium levels than non-survivors (Moghaddam et al., 2020). In addition, Coxsackievirus exhibits an increased mutation rate in selenium-deficient hosts, and infection with this virus reduces selenium levels in mice while enhancing viral replication (Jun et al., 2011; Molin et al., 2009). Selenium is essential for the human antioxidant system, and its deficiency impairs antioxidant capacity. Notably, the active site of GPX4 contains selenocysteine, indicating that selenium is necessary for synthesizing GPX4 and other reductases (Yao et al., 2021). Our research demonstrates that supplementing selenium in CVB3-infected mice upregulates GPX4 expression, thereby inhibiting CVB3-induced ferroptosis and suppressing viral replication.

## 5 Conclusion

Our results showed that CVB3 cleaves the P62 protein and further induces ferroptosis through the NRF2/GPX4 axis. CVB3-induced ferroptosis facilitates viral replication. P62 supplementation may inhibit virus-induced ferroptosis and thereby prevent CVB3 replication. Se supplementation inhibits CVB3 replication by upregulating the expression of GPX4 and suppressing the ferroptosis process.

## Data Availability

The datasets presented in this study can be found in online repositories. The names of the repository/repositories and accession number(s) can be found in the article/supplementary material.

## Footnotes

1
https://www.genome.jp/kegg/

2
https://ggplot2.tidyverse.org

3
https://cn.string-db.org/
